# Leprosy Investigation
*Journal of the Leprosy Investigation Committee. August, No. 1. Edited by Phineas S. Abraham, M.A., M.D., Secretary to the Committee. (London: Messrs. Macmillan and Co.)


**Published:** 1890-09-20

**Authors:** 


### Leprosy Investigation.*

It will be in recollection that the lamented death of Father Damien led to the formation of a committee, under the presidentship of H.R.H. the Prince of Wales, for the purpose of instituting measures for the investigation of the cause of leprosy, and for the relief of lepers. The inevitable dinner followed, which took place on January 13th, 1890, when the chair was taken by H.R.H. the Prince of Wales. It was decided to establish a national leprosy fund, and to appoint two specialists to visit India for the investigation of leprosy, but previous to their departure, to obtain as much information as possible from the most competent persons. Letters were therefore forwarded by the Secretary, the answers to which are contained in the first number of the journal of the leprosy committee. Remarks and suggestions as to points of investigation have been published in the order named, from Sir William Moore, K.C.I.E, Surgeon-General Beatson, Professor Cayley, of Netley, Dr. Patrick Manson, Surgeon-General J. Murray, Dr. Beavan Rake, Dr. C. Francis, Dr. V. C. Van der Straaten, Professor D. D. Cunningham, Surgeon-Major Pringle, Dr. Radcliffe Crocker, Dr. J. E. Payne, and Mr. Jonathan Hutchinson. Perhaps the most important point in connection with the subject is the question, if leprosy is contagious or not? on the side of communicability by contact or inoculation, we find Sir William Moore, Surgeon-General Beatson, Professor Cayley, Dr. Manson; while Dr. Rake, Dr. Francis, and Mr. Hutchinson do not accept contagion, and the other gentlemen named do not express an opinion. There is a similar divergence of opinion with regard to the question of heredity. Moore, Murray, Francis, and Rake (to a limited extent), believe leprosy to be hereditary. Beatson and Cayley doubt heredity as a cause. Mr. Jonathan Hutchinson is also against heredity. Mr. Hutchinson remarks, "If, however, we reflect a little on the possible modes of personal acquisition, it will be seen at once that insuperable fallacies underlie the facts which have been supposed to imply inheritance. If the disease can be easily produced by a small quantity of tainted food, who can feel

* Journal of the Leprosy Investigation Committee. August, No. 1. Edited by Phineas S. Abraham, M.A., M.D., Secretary to the Committee. (London: Messrs. Macmillan and Co.)

sure that in a leprosy district inheritance has taken any share?" That little word *if* is, however, as usual, a stumbling-block. It has yet to be proved that leprosy can be induced by a small quantity of tainted food. Of course, Mr. Hutchinson dwells much on his fish-eating theory. He says it is possible that fish may cause the disease in one of several ways. "First, it may be by the direct introduction of the bacillus into the stomach; secondly, it may be that some element in fish-food rouses the activity of a bacillus already existing in the tissues." And Mr. Hutchinson gives "arguments and facts" in support of the fish hypothesis. The arguments and facts amount to this: that it is not absolutely impossible that a person who is a leper may not have eaten fish some time in his life. Mr. Richardson considers salted fish as dangerous, or even more so, than fresh, for he says, "fish often contain bacilli of various species. It is known also, that the life of these parasites is assisted rather than otherwise by moderate salting." The fish theory, however, does not find much support from other contributors to the "Journal of the Leprosy Investigation Committee." Surgeon-General Francis remarks, "It will be advisable to thoroughly investigate the putrid fish theory. I have seen leprosy in Kumaon in the Himalayas, in no way differing from leprosy in Calcutta, and though fish, from its scarcity, is not an ordinary article of diet, it is available." Dr. Beavan Rake says: "We have only to imagine the leprosy bacilli thrown off from the bodies of those affected, and producing countless spores, which develop in water or soil, or in the bodies of some other animal," but he does not mention fish. Professor Cayley observes, "When at Gorruckpoor, I had under my observation a member of one of the families of Hindu bankers, who was a leper. From several generations one or more members of the family had suffered from leprosy. . . . . . This family was one of a caste of strict Hindus, that never touch fish, and one may say with certainty that for many generations no member of the family had ever eaten fish." Sir William Moore states "That fish diet is not the cause is evident from leprosy prevailing in the semi-desert districts of Western India, where the people never see fish, and by its not prevailing, especially among great fish eaters—Parsees, for example. Lepers flock from the country to the large towns of India—Bombay, for instance—where they have better chances of living by begging. This has become more the custom with increased facilities of communication. Hence the idea that leprosy is increasing among the fish-eating population, and hence a greater tendency to attribute it to a fish diet."

As regards preventive measures, Mr. Hutchinson asks the pertinent question, Can isolation be possibly carried out? And Mr. Hutchinson observes that the segregation of lepers never has been in any age, nor under any circumstances, more than a half measure. "The inhumanity of the procedure is such, that it has never been possible to prevent the healthy from mixing with the sick." Surgeon-General Beatson writes: "Segregation could not be made absolute and complete, without the life imprisonment of all lepers, and the separation of the sexes." Sir William Moore is even more emphatic on this point. This contributor says: "If segregation were possible in India it would only prevent communication of the disease by contact with leprous discharge. . . . It would not prevent persons, not apparently lepers, having leper children. . . . As a matter of fact, wholesale segregation in India is not possible, even putting aside the question of expense. . . . A person may have symptoms of leprosy in so slight a degree that difference of opinion as regards diagnosis would certainly arise, and no medical officer would consign such an individual to isolation. . . . The question may also be asked, whether it would be desirable to shut up a person in the first stage of leprosy in an asylum full of those suffering from an aggravated form of the disease? Again, the duty of collecting and guarding lepers must be confided to the police. The people would immediately fear that persons not lepers would be seized for various reasons, just as sane persons have been seized as lunatics in this country. . . I have repeatedly, both officially and otherwise, given advice against the wholesale segregation of lepers in India." As regards curative treatment, Dr. Radcliffe Crocker remarks that chaulmoogra oil is more efficacious in temperate climates, and gurjun oil in hot climates. Dr. Van der Straaten says he has known the treatment by chaulmoogra oil, as an inunction and internally, to have been very beneficial. Surgeon-General Murray found mudar *(calotropia gigantica)* the most efficacious medicine when used externally. Sir William Moore writes: "There is no remedial agent which directly affects leprosy. Under the influence of tonics, oils, nourishing diet, good personal hygiene, and general sanitation, improvement often takes place, and the progress of the disease may be perhaps temporarily arrested; the cachectic leper becomes a robust leper, but the leper remains a leper." A perusal of the remarks of the different contributors to the journal leaves the impression that while no one has yet been able to do more than theorize as to the cause and spread of leprosy, there is a greater consensus of opinion that sanitation in the broadest sense of the term is the best preventive measure. There are others besides Mr. Hutchinson who have attributed leprosy, if not to fish, to various kinds of putrid food. Dr. Francis thinks it is not improbable that the use of *lathyrus iativus*, a variety of pulse and of some other leguminous seeds, may give rise to something like leprosy. Sir William Moore quotes Mr. Collins' paper on leprosy, which appeared recently in the *Lancet*. Mr. Collins wrote "It is not that segregation is stamping out leprosy in Norway, but the increased material prosperity of the people; the growth of foreign trade, the inter communication with town life and the opportunities these give for better and more varied subsistence." Sir William Moore further points out that the dimniution and decline of leprosy in this country was contemporary with the advance of civilization. He quotes the Rev. Dr. Jessop's book, "Village Life in England Three Hundred Years Ago," and he remarks that the statements there made are applicable to Indian mofussil life of the present day. Of the natives of India he says:—"Born of a poorly-fed race, nursed by a semi-starved mother, working hard on food the use of which is semi-starvation, want of nutrition renders the system (of the Indian) prone to the attacks of any disease." And Sir William Moore expressly states that he has most confidence in the diminution of leprosy in India from the influence of advancing civilization and sanitation in the broadest sense of the term. "This includes the cleansing generally of towns and villages, drainage, ventilation, good water supply, the cheapening of salt, the prevention of scarcity, opposition to imprudent marriages, and measures for the prevention of syphilis."

We have only been able to touch on a few of the points connected with leprosy. But we especially refer to Dr. Patrick Manson's contribution, which treats mainly of the bacillus of leprosy, and which all interested in the subject should read. Dr. Beavan Rake's contribution, and his remarks on the transmission of the bacillus "through an intermediate host," should also demand attention. There is much in Surgeon-General Francis' paper of great interest. And Mr. Hutchinson's article evidences how much attention this eminent surgeon has paid to the subject. An appendix to the "Journal of the Leprosy Investigation Committee" contains a record of "the recent bibliography of leprosy." And our notice would not be satisfactory without bearing testimony to the able manner in which the journal has been edited by Dr. Phineas Abraham.

